# Neurobiological origin of spurious brain morphological changes: A quantitative MRI study

**DOI:** 10.1002/hbm.23137

**Published:** 2016-02-15

**Authors:** Sara Lorio, Ferath Kherif, Anne Ruef, Lester Melie‐Garcia, Richard Frackowiak, John Ashburner, Gunther Helms, Antoine Lutti, Bodgan Draganski

**Affiliations:** ^1^ LREN - Department of Clinical Neurosciences CHUV, University of Lausanne Lausanne Switzerland; ^2^ Wellcome Trust Centre for Neuroimaging, UCL Institute of Neurology, UCL London United Kingdom; ^3^ Department of Clinical Sciences Lund University, Medical Radiation Physics Lund Sweden; ^4^ Max Planck Institute for Human Cognitive and Brain Sciences Leipzig Germany

**Keywords:** quantitative MRI, MPRAGE, voxel‐based morphometry, gray‐matter volume, cortical thickness, in vivo histology, T1 mapping, T1‐weighted images

## Abstract

The high gray‐white matter contrast and spatial resolution provided by T1‐weighted magnetic resonance imaging (MRI) has made it a widely used imaging protocol for computational anatomy studies of the brain. While the image intensity in T1‐weighted images is predominantly driven by T1, other MRI parameters affect the image contrast, and hence brain morphological measures derived from the data. Because MRI parameters are correlates of different histological properties of brain tissue, this mixed contribution hampers the neurobiological interpretation of morphometry findings, an issue which remains largely ignored in the community. We acquired quantitative maps of the MRI parameters that determine signal intensities in T1‐weighted images (*R*_1_ (=1/T1), *R*_2_*, and PD) in a large cohort of healthy subjects (*n =* 120, aged 18–87 years). Synthetic T1‐weighted images were calculated from these quantitative maps and used to extract morphometry features—gray matter volume and cortical thickness. We observed significant variations in morphometry measures obtained from synthetic images derived from different subsets of MRI parameters. We also detected a modulation of these variations by age. Our findings highlight the impact of microstructural properties of brain tissue—myelination, iron, and water content—on automated measures of brain morphology and show that microstructural tissue changes might lead to the detection of spurious morphological changes in computational anatomy studies. They motivate a review of previous morphological results obtained from standard anatomical MRI images and highlight the value of quantitative MRI data for the inference of microscopic tissue changes in the healthy and diseased brain. *Hum Brain Mapp 37:1801–1815, 2016*. © **2016 The Authors. Human Brain Mapping Published by Wiley Periodicals, Inc.**

## INTRODUCTION

In the past two decades computational anatomy emerged as a useful tool for studying non‐invasively the healthy and diseased brain [Ashburner, 2009]. The computer‐based analysis of structural magnetic resonance imaging (MRI) data provides estimates of local brain volume, shape and cortical thickness that are indicative of underlying (patho)physiological processes [Ashburner et al., 2003; Burton et al., 2004; Fischl et al., 2002; Hibar et al., 2015; Rektorova et al., 2014; Ryan et al., 2013; Qiu et al., 2014]. From a simplistic neurobiological point of view, a loss in gray matter volume or cortical thickness is interpreted as a sign of neuronal loss, while an increase is considered as a correlate of use‐dependent brain plasticity [Draganski et al., 2014; Zatorre, 2013]. While issues concerning MRI data processing and statistical analysis have been largely resolved [Draganski and Kherif, 2013; Thomas and Baker, 2013], morphological brain changes detected from standard (e.g. T1‐weighted) anatomical MRI data may reflect true macroscopic morphological brain changes or may be the spurious results of biophysical processes taking place at the microstructural scale [Weiskopf et al., 2015]. The characterization of the latter processes—of primary interest in neuroscience research—requires specific MRI biomarkers of brain tissue microstructure.

Visual inspection of MRI and histological data illustrates the high correlation between MRI contrast and region‐specific degrees of myelination [Fatterpekar et al., 2002; Fukunaga et al., 2010; Geyer et al., 2011]. Quantitative MRI (qMRI) provides estimates of the parameters of the MRI signal that are valuable biomarkers of brain tissue microstructure [Geyer and Turner, 2011]. A high correlation between iron concentration and the effective transverse relaxation rate *R*_2_* (=1/T2*) has been observed in ferritin‐rich structures [Gelman et al., 1999; Langkammer et al., 2010; Yao et al., 2009]. The dominant contribution of myelin to the parameter *R*_1_ (=1/T1) has been also been established [Rooney et al., 2007] except in subcortical brain areas with high levels of iron [Helms et al., 2009; Lorio et al., 2014]. Multivariate analysis have provided the most compelling evidence for the relationship between MRI parameters and tissue microstructure over the entire brain [Callaghan et al., 2015a]. Beyond this empirical evidence, current efforts are shedding a more detailed and comprehensive light on this relationship with the aim to characterize tissue microstructure from MRI data—in vivo histology [Dinse et al., 2015; Stüber et al., 2014]. The combination of MRI data with different contrast mechanisms—each drawing on complementary features of tissue microstructure, may prove to be an essential step towards that goal [Mohammadi et al., 2015; Stikov et al., 2015]. Whole‐brain high resolution qMRI maps have allowed myeloarchitectonic studies of the cerebral cortex in vivo at 3 T and 7 T, highlighting densely myelinated primary and extrastriate visual areas exhibiting a high degree of overlap with topological fMRI maps [Cohen‐Adad, 2014; Dick et al., 2012; Lutti et al., 2014; Sereno et al., 2013]. The remarkable similarity of the changes in *R*_1_ values across the cortical layer with histological data highlights the sensitivity of qMRI to subtle variations in myeloarchitecture, particularly at high field strength, offering promising perspectives for the parcellation of the cerebral cortex from in vivo MRI data [Lutti et al., 2014; Waehnert et al., 2016]. In conjunction with image segmentation, image registration and intra‐cortical surface extraction techniques that draw on the microstructural information provided by qMRI [Bazin et al., 2014; Tardif et al., 2015b; Waehnert et al., 2014, 2016], the combination of high‐resolution quantitative and functional MRI data opens new perspectives for the study of brain structure [Helbling et al., 2015; Olman et al., 2012; Polimeni et al., 2010; Turner and Geyer, 2014].

qMRI provides quantitative and specific biomarkers of tissue microstructure with enhanced sensitivity to the biophysical changes taking place in the healthy and diseased brain [Deoni et al., 2008; Focke et al., 2011; Tardif et al., 2015a; Weiskopf et al., 2013]. qMRI data has been employed for the study of pathological conditions such as multiple sclerosis [Khalil et al., 2015; Louapre et al., 2015], spinal cord injury [Freund et al., 2013] and Alzheimer's Disease [Langkammer et al., 2014]. The establishment of normative qMRI values for the healthy and diseased brain motivates the characterization with qMRI of brain changes associated with healthy ageing, highlighting the associated demyelination and iron deposition processes [Callaghan et al., 2014; Draganski et al., 2011; Ghadery et al., 2015; Lorio et al., 2014]. Recent technological advances allowing a reduction of the acquisition time [Langkammer et al., 2015; Xu et al., 2013] and of the impact of subject motion [Callaghan et al., 2015b; Zaitsev et al., 2006] are expected to facilitate the use of qMRI techniques on clinical populations.

Despite the benefits of qMRI highlighted above, standard anatomical MRI data (e.g., T1‐weighted, T2‐weighted…) remain the workhorse of the majority of computational neuroscience studies. While the contrast in standard anatomical MRI images is mainly driven by one MRI parameter, contributions from other parameters are also present. The dependence of these MRI parameters on different histological tissue properties hinders the interpretation at the microstructural level of computational anatomy results obtained from standard anatomical images. The aim of this study is to illustrate this lack of specificity and to demonstrate how microstructural processes in brain tissue might lead to the spurious detection of tissue volume changes in morphometry studies. Here we will focus on magnetization‐prepared‐rapid‐gradient‐echo (MPRAGE) T1‐weighted (T1w) data, which has been extensively used due to its high gray‐white matter contrast at high image resolution [Mugler and Brookeman, 1990]. While the contrast in MPRAGE images is predominantly driven by T1, proton density (PD − concentration of MRI‐observable water) and *R*_2_* also have an impact described by the MPRAGE signal equations (e.g. [Deichmann et al., 2000; Helms et al., 2008a]). We acquired whole‐brain quantitative maps of *R*_1_, *R*_2_*, and PD in a large cohort of healthy subjects. Using the analytical expression of the MPRAGE signal we computed synthetic T1w images based on subsets of these maps [Deichmann et al., 2000; Nöth et al., 2015]. Manipulation of the contrast in synthetic T1w images has been used recently for the visualisation of brain tumours [Nöth et al., 2015]. Improved visibility of tumours was shown from synthetic images computed from the parameter T1 only, highlighting the relationship between tissue microstructure and MRI contrast. We extend this approach to the quantitative analysis of the neurobiological changes underlying apparent volume changes in morphometric studies. The synthetic images were processed using identical state‐of‐the art computational anatomy algorithms to obtain voxel‐based measures of gray matter volume or vertex‐based estimates of cortical thickness. We investigated the effect of the MRI parameters on the estimation of volume and thickness in the framework of voxel‐based morphometry (VBM) and surface‐based analysis.

## MATERIAL AND METHODS

### Subjects

One hundred twenty healthy adults (56 men, age range 18–78 years, mean 39 ± 16 years), (64 women, age range 18–85 years, mean 40 ± 19 years) were examined on a 3 T whole‐body MRI system (Magnetom Prisma, Siemens Medical Systems, Germany), using a 64‐channel RF receive head coil and body coil for transmission. On visual inspection study participants showed neither macroscopic brain abnormalities, i.e. major atrophy, nor signs of overt vascular pathology—i.e. microbleeds and white matter lesions. Participants with extended atrophy or with white matter hyperintensities (WMH) of grade 2 or more by the Scheltens rating scale [Scheltens et al., 1993] were not included. Informed written consent was obtained prior to study according to the approval requirements of the local Ethics committee.

### Data Acquisition

The whole‐brain protocol for quantitative mapping of *R*_1_, *R*_2_*, and PD comprised two multiecho 3D fast low angle shot (FLASH) acquisitions [Helms et al., 2008a, 2009; Weiskopf et al., 2013], one radio frequency (RF) transmit field map and one static magnetic (B0) field map [Lutti et al., 2010, 2012]. The FLASH datasets were acquired with predominantly proton density‐weighted (PDw) and T1w contrast with appropriate choice of repetition time (TR) and flip angle (*α*) (PDw: TR/*α* = 24.5 ms/6°; T1w: TR/*α* = 24.5 ms/21°). Multiple gradient echoes were acquired for each FLASH acquisition with alternating readout polarity at eight equidistant echo time (TE) between 2.34 ms and 18.72 ms. The image resolution was 1 mm isotropic, the field of view was 256 × 240 × 176 mm and the matrix size—256 × 240 × 176. Parallel imaging was used along the phase‐encoding (PE) direction (acceleration factor 2 GRAPPA reconstruction [Griswold et al., 2002]), 6/8 partial Fourier was used in the partition direction. Three‐dimensional echo‐planar imaging (EPI) spin‐echo (SE) and stimulated echo (STE) images were used to calculate maps of the transmit field B1+ [Lutti et al., 2010, 2012] and correct for the effect of RF transmit inhomogeneities on the quantitative maps [Helms et al., 2009; Helms and Dechent, 2009; Weiskopf et al., 2013]. To correct the RF transmit field maps for geometric distortion and off‐resonance effects, a map of B0 was acquired using a two‐dimensional double‐echo FLASH sequence [Lutti et al., 2010, 2012]. The total acquisition time was 18 min.

Calculation of the quantitative maps from the acquired data was implemented with the Voxel‐Based Quantification toolbox [Draganski et al., 2011] running under SPM12 (Wellcome Trust Centre for Neuroimaging, London, UK; http://www.fil.ion.ucl.ac.uk/spm) and Matlab 7.11 (Mathworks, Sherborn, MA, http://www.mathworks.com). The *R*_2_* maps were calculated from the regression of the log‐signal of the eight PD‐weighted (PDw) echoes. The *R*_1_ and PD maps were computed as described in [Helms et al., 2008a], using the PDw and T1w images with minimal echo time (TE = 2.34 ms) in order to minimize *R*_2_* bias on the *R*_1_ and PD estimates. The *R*_1_ maps were corrected for local RF transmit field inhomogeneities [Lutti et al., 2012] and imperfect RF spoiling using the approach described by [Preibisch and Deichmann, 2009].

#### Synthetic MPRAGE T1‐weighted images

Synthetic MPRAGE images were created from the acquired *R*_1_, PD, and *R*_2_* maps using the MPRAGE signal equation [Deichmann et al., 2000; Nöth et al., 2015]:

(1) $$T1w\left(R1,{PD,R2}^{*}\right)=\;PD\cdot \sin\left(\alpha \right)\cdot \exp\left(-TE\cdot R_{2}^{*}\right)\cdot \left(E_{4}\cdot \frac{1-2{\cdot E}_{1}+E_{1}\cdot E_{2}}{1+E_{1}\cdot E_{2}\cdot E_{3}}+T_{1}^{*}{\cdot R}_{1}\cdot \frac{1+E_{1}\cdot E_{2}\cdot E_{3}-E_{4}-E_{4}{\cdot E}_{1}\cdot E_{2}}{1+E_{1}\cdot E_{2}\cdot E_{3}}\right)$$

$${with\;T}_{1}^{*}={\left(R_{1}-\frac{1}{ES}\cdot ln(cos(\alpha ))\right)}^{-1};\;E_{1}=exp\left(-TI{\cdot R}_{1}\right);E_{2}=exp\left(-TD\cdot R_{1}\right);\;E_{3}=exp\left(-\frac{\tau }{T_{1}^{*}}\right);\;E_{4}=exp\left(-\frac{\tau }{2\cdot T_{1}^{*}}\right)$$

where *α* (nominal RF excitation flip angle), TE, ES (echo spacing), TI (inversion time), *τ* (readout duration), and TD (delay time) are parameters of the simulated MPRAGE acquisition. PD, $R_{2}^{*}$, and *R*_1_ are the MRI parameters of brain tissue and are provided by the quantitative maps described above.

The acquisition parameters of the synthetic MPRAGE images were set according to [Tardif et al., 2009] to yield optimal gray‐white matter contrast (*α* = 9°; ES = 9.9 ms; TI = 960 ms; *τ* = 176 × ES; TR = 2,420 ms).

Equation (1) represents the product of three terms that contain the contribution of each MRI parameter to the synthetic images:

$$f\left(PD\right)=PD$$

$$f\left({R2}^{*}\right)=exp(-TE\cdot {R2}^{*})$$

$$f\left(R1\right)=E_{4}\cdot \frac{1-2{\cdot E}_{1}+E_{1}\cdot E_{2}}{1+E_{1}\cdot E_{2}\cdot E_{3}}+T_{1}^{*}{\cdot R}_{1}\cdot \frac{1+E_{1}\cdot E_{2}\cdot E_{3}-E_{4}-E_{4}{\cdot E}_{1}\cdot E_{2}}{1+E_{1}\cdot E_{2}\cdot E_{3}}$$

Each of these contributions may be removed from the synthetic T1w images by setting the corresponding term to 1. The synthetic images used in the current study are listed in Table 1 with the MRI parameters used in the corresponding signal equation.

**Table 1:** Types of synthetic MPRAGE images computed from the maps of MRI parameters using the corresponding signal equation

MPRAGE synthetic image	MRI parameters	Equation
T1w(*R*_1_)	*R*_1_	$f\left(R1\right)\cdot \sin\left(\alpha \right)$
T1w(*R*_1_,PD)	*R*_1_, PD	$f\left(R1\right)\cdot f\left(PD\right)\cdot \sin\left(\alpha \right)$
T1w(*R*_1_,*R*_2_*)	*R*_1_, R_2_*	$f\left(R1\right)\cdot f\left(R_{2}^{*}\right)\cdot \sin\left(\alpha \right)$
T1w(*R*_1_,PD,*R*_2_*)	*R*_1_, PD, *R*_2_*	$f\left(R1\right)\cdot f\left(PD\right)\cdot f\left(R_{2}^{*}\right)\cdot \sin\left(\alpha \right)$

### Data Processing

#### Gray matter estimates—Voxel‐based morphometry

For VBM analysis, all synthetic MPRAGE T1w images were processed independently with the same default settings and classified into different tissue classes: gray matter (GM), white matter (WM), cerebral‐spinal fluid (CSF) and non‐brain tissue, using the “unified segmentation” approach in SPM12 [Ashburner and Friston, 2005]. Aiming at optimal anatomical precision we applied the diffeomorphic registration algorithm DARTEL [Ashburner, 2007]. The warped GM probability maps derived from the synthetic MPRAGE T1w images were scaled by the Jacobian determinants of the deformation fields to account for local compression and expansion due to linear and nonlinear transformation [Ashburner and Friston, 2000]. The GM maps were then smoothed by convolution with an isotropic Gaussian kernel of 6 mm full‐width‐at‐half‐maximum (FWHM).

#### Cortical thickness estimates—Surface‐based analysis

For surface‐based analysis all synthetic MPRAGE T1w images were processed independently with the same default settings to measure cortical thickness using the open source FreeSurfer package (http://surfer.nmr.mgh.harvard.edu/). Briefly, image processing included removal of non‐brain tissue using a hybrid watershed/surface deformation procedure [Ségonne et al., 2004], automated Talairach transformation, extraction of the subcortical WM and deep GM structures (including hippocampus, amygdala, caudate, putamen, ventricles) [Fischl et al., 2004], intensity normalisation [Sled et al., 1998] and tessellation of the GM/WM boundary. The GM/WM and GM/CSF borders were placed where the highest intensity gradients defined the transition to the other tissue class [Fischl and Dale, 2000; Ségonne et al., 2007]. Cortical thickness was calculated as the shortest distance from the GM/WM boundary to the GM/CSF boundary at each vertex on the tessellated surface [Fischl and Dale, 2000]. The cortical thickness maps were warped to standardised space and smoothed by convolution with an isotropic Gaussian kernel of 15 mm FWHM.

### Statistical Analysis

#### Main effect of MRI parameters

Gray matter volume and cortical thickness were analysed separately using a mass‐univariate approach and flexible factorial design. The morphometric features extracted from the different types of synthetic MPRAGE T1w images (T1w(*R*_1_), T1w(*R*_1_,PD), T1w(*R*_1_, *R*_2_*), T1w(*R*_1_,PD, *R*_2_*)) were included into the design matrix in separate columns that indicated the type of synthetic T1w data used. Regional differences were examined creating voxel‐wise or vertex‐wise statistical parametric maps using the General Linear Model (GLM) and the Random Field Theory implemented in SPM for the VBM analysis, and SurfStat for the surface based analysis (http://www.math.mcgill.ca/keith/surfstat/). One‐tailed *T*‐statistic was computed to detect differences over a whole‐brain search volume. The search volume for the GM estimates was defined using the automated anatomical labelling (AAL), human brain atlas [Tzourio‐Mazoyer et al., 2002], the (SUIT) atlas of cerebellum and brainstem [Diedrichsen, 2006] and the basal ganglia human area template (BGHAT) [Prodoehl et al., 2008]. We applied a statistical threshold at *P* < 0.05 after family‐wise error (FWE) correction for multiple comparisons over the whole search volume or surface.

#### Linear and nonlinear age effect

To investigate age‐dependent local effects on GM volume and cortical thickness, the concatenated morphometric features were also split into two design matrices—one for GM volume and the other for cortical thickness including age and gender as additional variables. We used a polynomial model to identify voxels/vertices in which the variance in GM volumes and cortical thickness was better explained by a quadratic rather than a linear function of age. The model included regressors for the quadratic and original age values, which were mean centred. Recent studies on brain aging that included nonlinear analysis supported the use of polynomial models up to degree 3 [Walhovd et al., 2011] because polynomials with higher degrees or exponential functions can be highly accurate.

### Gray‐White Matter Contrast Analysis

To investigate the variation of morphological measures across synthetic T1w images, we used the image contrast between white and gray matter contrast calculated as follows:

(2) C=WMInt−GMInt(WMInt+GMInt)2

where WM_Int_ and GM_Int_ are the WM and GM intensities of a given synthetic T1w image. We measured WM intensity at 1 mm subjacent to the gray‐white matter border along the surface normal and we sampled GM intensity at 35% through the thickness of the cortical ribbon, normal to the GM‐WM border. The 35% sampling procedure allowed us to be conservatively close to the GM‐WM border and to adjust the sampling distance in regions of low cortical thickness (as opposed to using a constant value across the entire border which could be problematic for thinner cortical areas). This sampling procedure has been used previously to study contrast change in ageing [Salat et al., 2009].

We used a linear model to investigate the correlation between the cortical thickness changes and the variation of gray‐white matter contrast across synthetic T1w MPRAGE images. The model was implemented vertex‐wise according to the following equation:

(3) ΔCt=β·ΔC+ ε

where ΔCt and Δ*C* are respectively the changes in cortical thickness and GM‐WM contrast between synthetic T1w images, *β* is the linear coefficient estimated for every vertex, and *ε* represents the residuals of the model. Both Δ*C* and ΔCt were smoothed by convolution with an isotropic Gaussian kernel of 15 mm FWHM. To assess the quality of parameter estimation we computed *T*‐values for the linear coefficient, testing against the null hypotheses that *β* was equal to zero. The statistical significance level was set at *P*_FWE_ < 0.05.

The variations across synthetic images of the contrast calculated using Eq. (2) were examined by comparison with the theoretical predictions obtained from the signal equations. For example the contrast change between T1w(*R*_1_) and T1w(*R*_1_, PD) images can be derived from Eq. (1) and (2):

(4) $$C_{T1w(R1)}-C_{T1w\left(R1,PD\right)}=\frac{4\left(R_{PD}-1\right)R_{T1w(R1)}}{\left(1+R_{PD}\right)\left(1+R_{T1w(R1)}\right)}$$

where *R*_PD_ and *R*_T1w(_ *R*_1)_ are the ratio between gray and white matter intensity of the PD and T1w(*R*_1_) values respectively. Equation (4) lays down the relationship between the contrast variation across the types of synthetic images and the MRI‐histological properties of the tissue. It may therefore be used to infer the microstructural mechanisms driving an observed contrast change. The white and gray matter values were sampled according to the aforementioned procedure for contrast estimation [Eq. (2)].

## RESULTS

### MRI Parameters Main Effects

**Figure 1:**
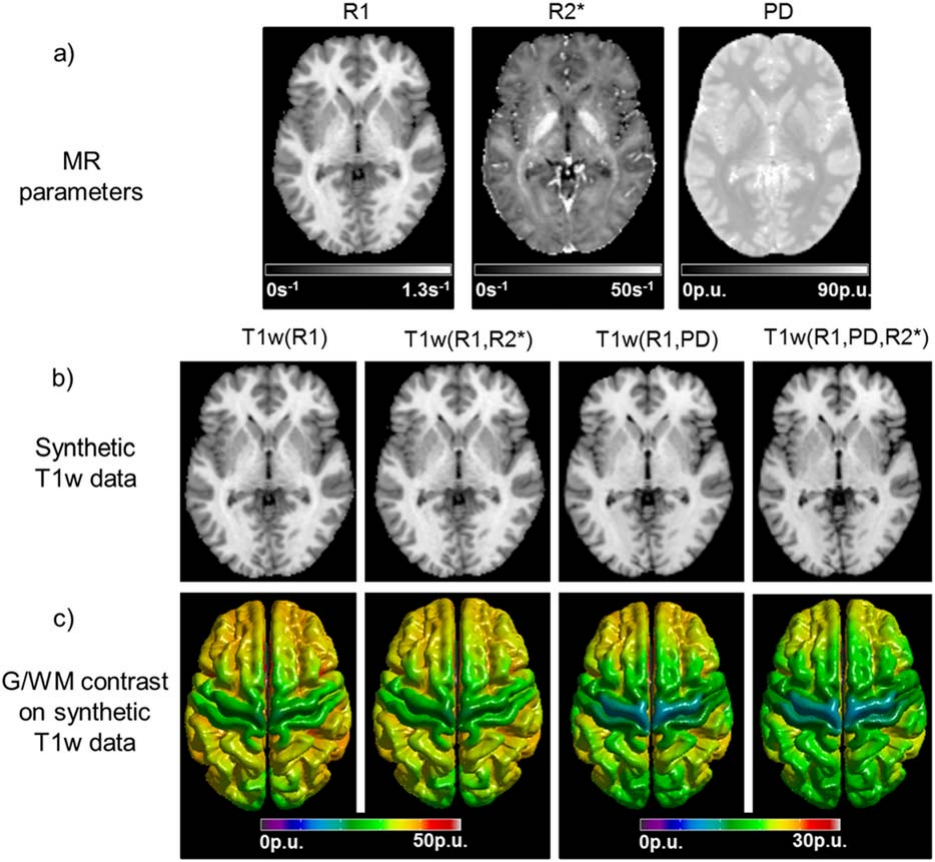
Example of synthetic MPRAGE T1w images. Individual quantitative maps of the MRI parameters R1, PD and R2* (a). Synthetic T1w MPRAGE images computed from the maps of MRI parameters and the MPRAGE signal equation [Eq. (1)] (b). Gray‐white matter contrast in the synthetic T1w images calculated from Eq. (2) (c). [Color figure can be viewed in the online issue, which is available at http://wileyonlinelibrary.com.]

Figure 1 shows an example set of synthetic T1w images calculated from the acquired quantitative MRI data. On visual inspection putamen, pallidum, and thalamus show higher contrast against the surrounding tissue on the T1w(*R*_1_, *R*_2_*) compared with the other synthetic images (see Fig. 1b). As shown by Figure 1c, the sensorimotor and the visual cortex exhibited higher contrast on T1w(*R*_1_) and T1w(*R*_1_, *R*_2_*) images than on T1w(*R*_1_,PD) and T1w(*R*_1_,PD, *R*_2_*), highlighting the role of PD in reducing GM‐WM contrast in these regions.

**Figure 2:**
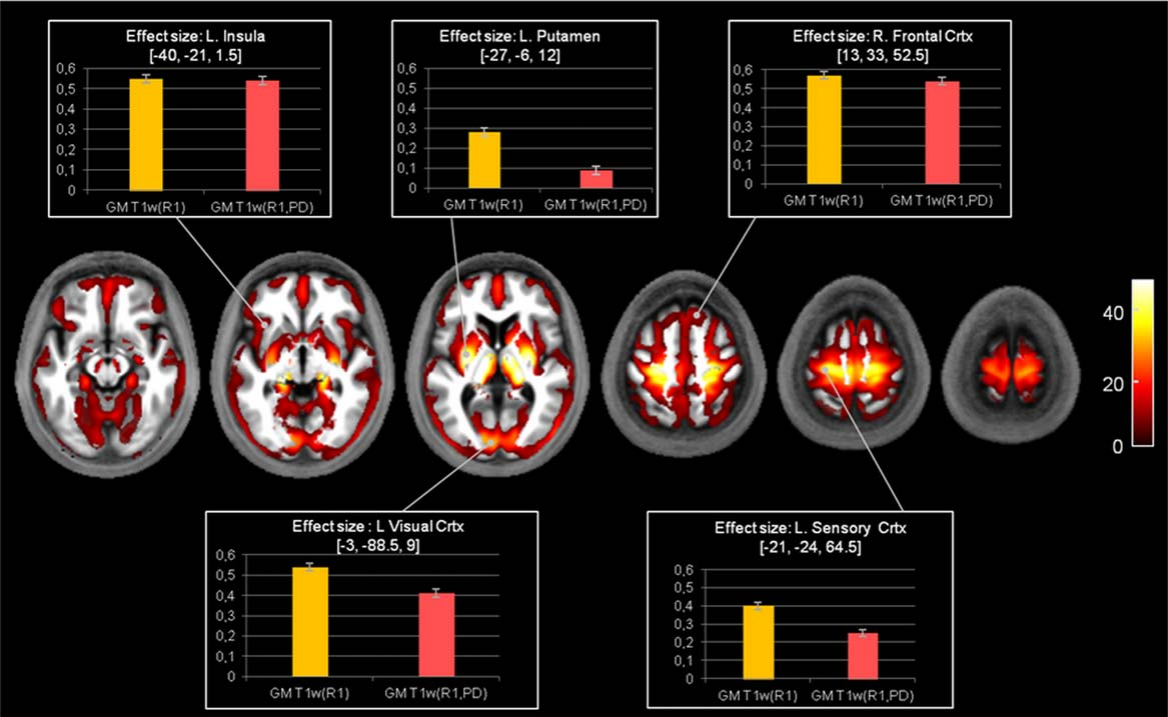
Pattern of higher gray matter volume estimation from T1w(R1) compared with T1w(R1,PD) synthetic images (*P*_FWE_ < 0.05). The bar plots represent the effect size of the paired *t*‐test. [Color figure can be viewed in the online issue, which is available at http://wileyonlinelibrary.com.]

The GM volumes extracted from T1w(*R*_1_) images were significantly higher than those obtained from T1w(*R*_1_,PD) images in the thalamus, the dorsolateral part of putamen, the substantia nigra and over the entire cortical ribbon (see Fig. 2). We note that despite the widespread significant results covering the whole cortex, there were local differences in *T*‐values and effect size in the sensorimotor and the visual cortex (see Fig. 2) consistent with the reduction in regional image contrast due to PD (Fig. 1c).

**Figure 3:**
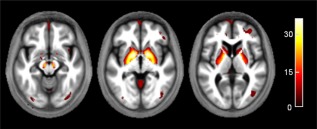
Higher gray matter volume estimation from T1w(*R*_1_,*R*_2_*) images compared with T1w(*R*_1_). The statistical map of a paired *t*‐test is retrieved at a threshold of *P*_FWE_ < 0.05 and displayed in standard MNI space. [Color figure can be viewed in the online issue, which is available at http://wileyonlinelibrary.com.]

Our statistical analysis of the impact of the *R*_2_* parameter on GM volume estimates showed higher GM volumes from T1w(*R*_1_, *R*_2_*) compared with T1w(*R*_1_) images in the pallidum, dorsoventral part of the putamen and substantia nigra (Fig. 3)—consistent with the enhancement of image contrast due to *R*_2_* in these regions (Fig. 1b).

When estimating the combined effect of PD and *R*_2_* on the GM volumes extracted from the T1w images the GM volumes estimates from T1w(*R*_1_) were higher compared with T1w(*R*_1_, PD, *R*_2_*) images (Fig. 8). We found a regional pattern similar to the effects of the PD parameter (Fig. 2), with the highest volumetric differences located in the deep brain nuclei, the sensorimotor, and visual cortex.

**Figure 4:**
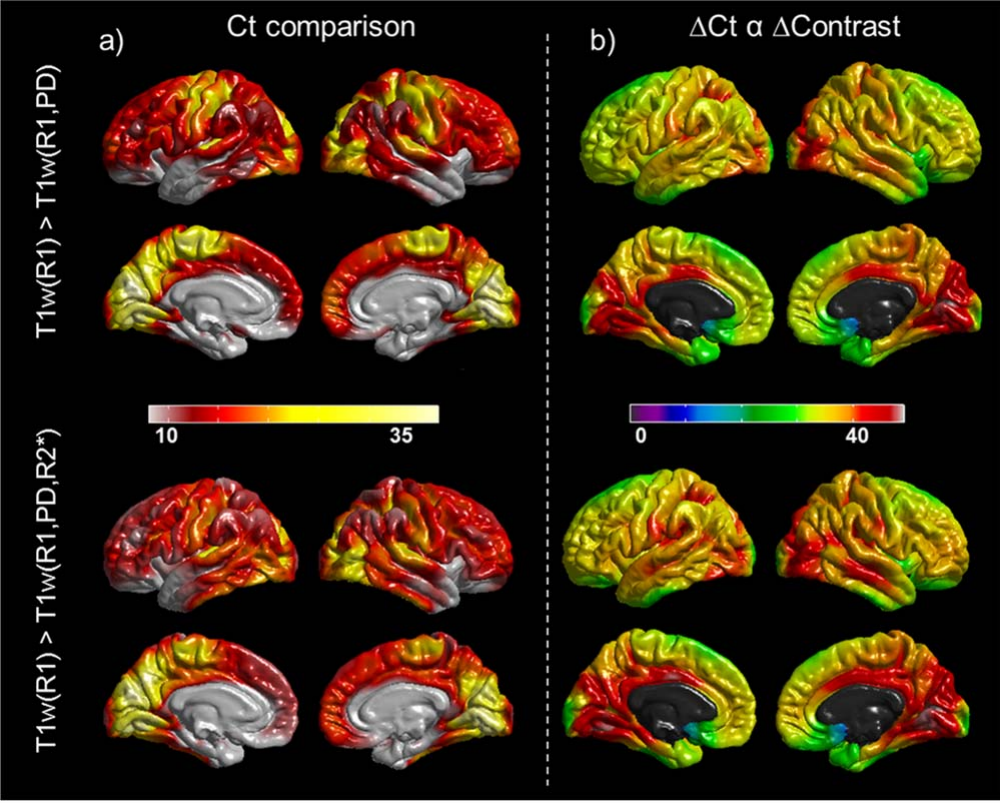
Statistical comparison of cortical thickness estimates obtained from different synthetic image modalities (*P*_FWE_ < 0.05) (**a**). Correlation between the changes in cortical thickness across modalities and the corresponding change in image contrast (**b**). The top row compares results from T1w(*R*_1_) and T1w(*R*_1_,PD) images and the bottom row compares T1w(*R*_1_) and T1w(*R*_1_,PD, *R*_2_*) images. [Color figure can be viewed in the online issue, which is available at http://wileyonlinelibrary.com.]

The sensorimotor and visual cortex showed higher cortical thickness estimates from T1w(*R*_1_) than T1w(*R*_1_, PD) and T1w(*R*_1_, PD, *R*_2_*) images (see Fig. 4a). These cortical thickness changes were positively correlated with the variation of image contrast between the corresponding T1w images (see Fig. 4b).

No differences in cortical thickness were found between T1w(*R*_1_) and T1w(*R*_1_, *R*_2_*) images.

### Gray‐White Matter Contrast Analysis

**Figure 5:**
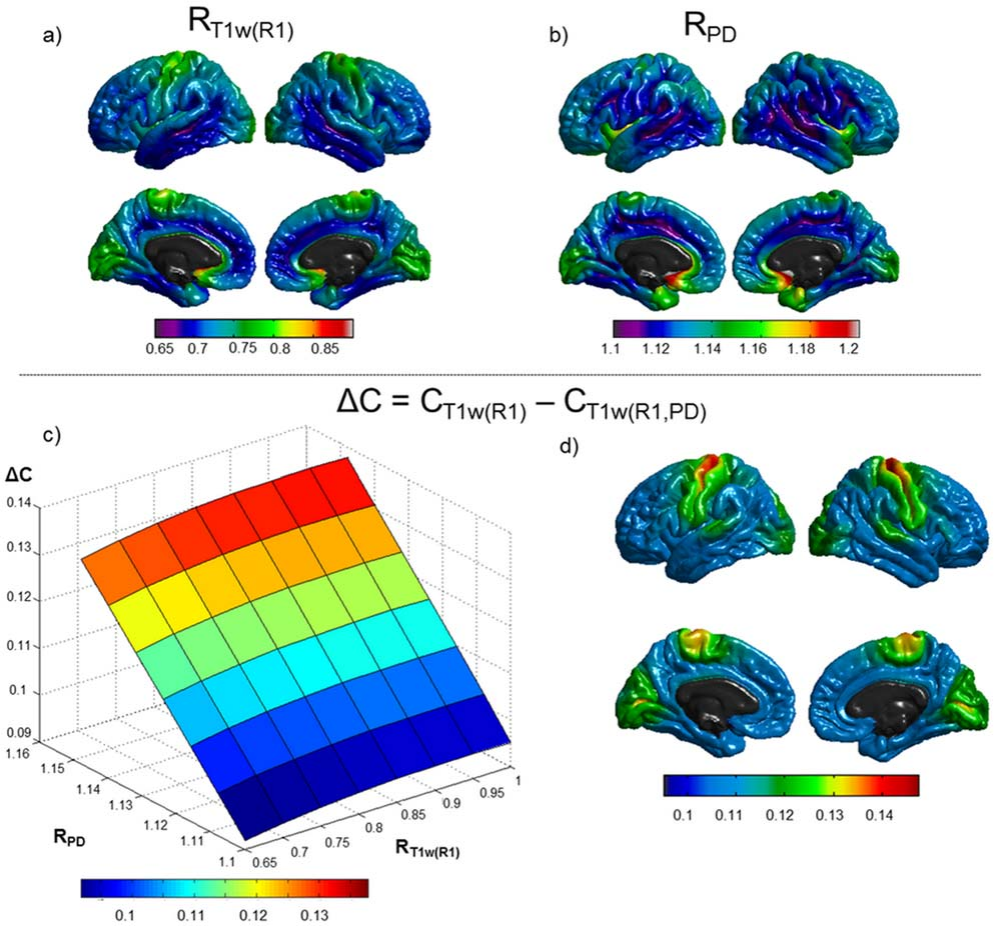
Gray‐white matter ratios of T1w(*R*_1_) (**a**) and proton density (PD) (**b**). Change in gray‐white matter contrast due to proton density predicted by Eq. (4) (**c**). Change in gray‐white matter contrast due to proton density computed from the T1w(*R*_1_) and T1w(*R*_1_,PD) synthetic images (**d**). [Color figure can be viewed in the online issue, which is available at http://wileyonlinelibrary.com.]

We found higher *R*_T1w(_ *R*_1)_ and R_PD_ values in the sensorimotor and visual cortex (see Fig. 5a,b), mainly due to the higher *R*_1_ and lower PD values in these GM regions. Figure 5c shows the contrast change between T1w(*R*_1_) and T1w(*R*_1_,PD) images predicted by Eq. (4) for the range of PD and T1w(*R*_1_) values present in the brain. *R*_PD_ produces a higher contrast change than *R*_T1w(_ *R*_1)_. These theoretical predictions were in good agreement with the contrast change calculated from the synthetic data (Fig. 5d). The regions of highest contrast reduction were the sensorimotor and visual cortex (Fig. 5d), consistent with the GM volume (Fig. 2) and cortical thickness (Fig. 4) reductions due to the inclusion of PD.

### Age Main Effect

**Figure 6:**
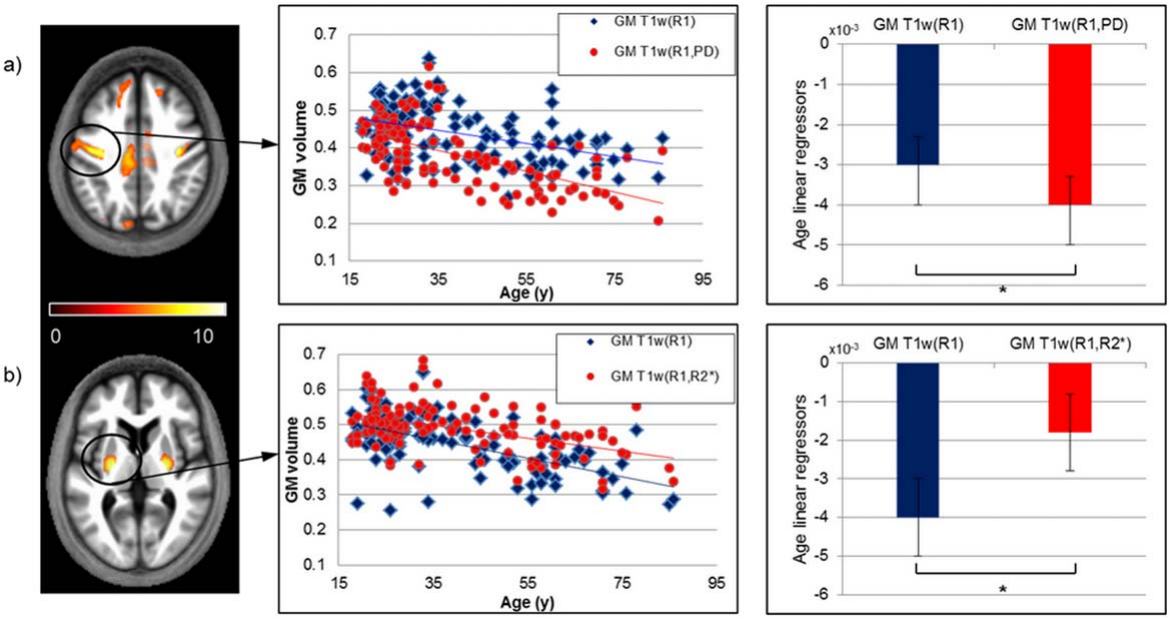
Interaction between age‐related linear reduction in gray matter volume and T1w(*R*_1_) and T1w(*R*_1_,PD) (**a**) and T1w(*R*_1_) and T1w(*R*_1_, *R*_2_*) (**b**). [Color figure can be viewed in the online issue, which is available at http://wileyonlinelibrary.com.]

As repeatedly reported in the literature, we found significant (*P*_FWE_ < 0.05) age‐related linear GM volume reductions primarily in frontal regions and within the ventral part of the putamen. While this effect was common to all T1w synthetic images, we observed significant interactions between age and image modality (see Fig. 6): we report stronger age‐related GM volume reduction in the sensorimotor cortex from T1w(*R*_1_,PD) compared with T1w(*R*_1_) images (see Fig. 6a). Similarly, a higher gray matter volume loss associated with age was obtained from T1w(*R*_1_,PD,*R*_2_*) with respect to the estimates derived from T1w(*R*_1_) images in the aforementioned regions. The dorsal part of the putamen and the pallidum showed stronger age‐related GM volume reduction estimated from T1w(*R*_1_) when compared with estimates from T1w(*R*_1_,R2*) images (see Fig. 6b). The same subcortical regions also showed enhanced GM volume loss associated with age, when the T1w(*R*_1_)‐based estimates were compared with T1w(*R*_1_,PD, *R*_2_*)‐based ones.

The obtained GM volumes showed significant negative correlations with the quadratic age term in the hippocampus and insula. No significant interaction was found between these correlations and the type of synthetic T1w images examined.

**Figure 7:**
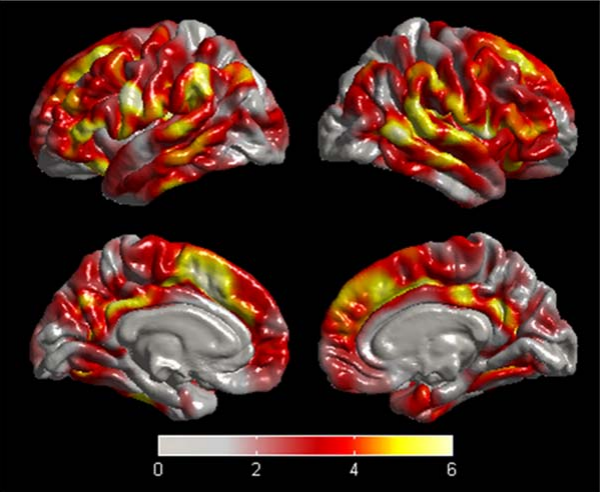
Statistical parametric map of the age‐associated cortical thickness decrease at *P*_FWE_ < 0.05. The regression between age and cortical thickness was identical for all image modalities. [Color figure can be viewed in the online issue, which is available at http://wileyonlinelibrary.com.]

The linear regression between cortical thickness estimates and subject age showed significant (*P*_FWE_ < 0.05) negative correlation in the superior‐frontal and lateral cortex (see Fig. 7). This correlation was valid for the cortical thickness computed from all synthetic T1w images. There was no interaction between linear age effect and image modalities used to estimate the cortical thickness.

**Figure 8:**
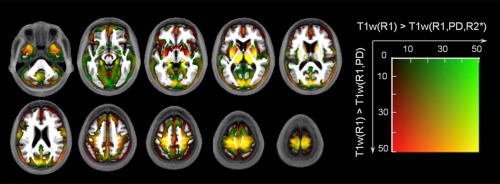
Pattern of higher gray matter volume estimation from T1w(*R*_1_) compared with T1w(*R*_1_,PD) and to T1w(*R*_1_,PD,*R*_2_*) synthetic images. The *t* score (*P*_FWE_ < 0.05) for the combined effects is indicated by the colour square. [Color figure can be viewed in the online issue, which is available at http://wileyonlinelibrary.com.]

We did not observe any significant correlation between the quadratic age term and cortical thickness for any synthetic T1w modality examined.

## DISCUSSION

Our study highlights the differential impact of brain tissue histological properties on the estimates of apparent gray matter volume and cortical thickness obtained from T1w images. We report significant changes in gray matter volume and cortical thickness when the contributions of myelin, iron, and tissue water concentration were included—via their surrogate MRI biomarkers—in the anatomical images used for extraction of the morphological estimates. Significant differences were also observed when changes in brain morphology due to ageing were considered. These results are illustrations of the effects of microstructural processes on brain morphology measures and are highly relevant for morphometry findings commonly reported in computational neuroscience studies. These results emphasize a need for critical reappraisal of previous neurobiological interpretations of computational anatomy findings and highlight the benefits of qMRI data for the study of the biophysical processes taking place in the healthy and diseased brain.

### Cortical Findings

The first major finding of this study is the specific pattern of differences in GM volume and cortical thickness obtained from different subsets of synthetic T1w images derived from the very same study population and processed with identical default settings. These symmetric changes included the primary sensorimotor and visual cortex and proved to be highly correlated with the corresponding variation of image contrast between GM and WM.

The inclusion of PD (i.e. observable water content) in the signal equation of the synthetic T1w images led to a reduction in the gray matter volume and cortical thickness. While present over most of gray matter, this effect was particularly pronounced in early myelinating regions such as sensorimotor and visual cortex, which exhibit high values for *R*_1_—a correlate of myelin concentration [Lutti et al., 2014; Sereno et al., 2013]. We demonstrated that these morphometric differences are due to a higher ratio of the PD values between gray and white matter in these areas—in‐line with the known inverse relationship between *R*_1_ and PD [Helms et al., 2008a;Van de Moortele et al., 2009].

Previous studies have reported differences in iron content between cortical GM layers II/III and WM in early myelinated regions [Langkammer et al., 2010; Stüber et al., 2014]. Given the known relationship between iron concentration and *R*_2_*, our hypothesis was that the inclusion of *R*_2_* in the MPRAGE signal equation would be associated with GM volume or cortical thickness change [Yao et al., 2009]. We attribute the absence of such observation to the weak impact of *R*_2_* on MPRAGE images due to their short echo times (TE). The influence of *R*_2_* could only be detected in the basal ganglia, which exhibit high *R*_2_* values due to their high iron content [Yao et al., 2009]. Note that this effect was counterbalanced by the PD values when all MRI parameters involved in the contrast of MPRAGE images were considered.

#### Modulation of cortical findings by age

The demonstrated age‐dependent loss in cortical volume and thickness is consistent with quantitative stereological analyses on post mortem specimens showing a 10% reduction of neocortical neurons with relative preservation of neuronal size and synaptic density [Pakkenberg et al., 2003]. The observation of morphological changes in synthetic images computed only from the MRI parameter *R*_1_—an MRI biomarker of myelin content—points towards a decrease of GM volume and cortical thickness driven by a reduction of myelin content that could reflect neuronal death.

Importantly, we show a differential estimation of age‐associated GM volume loss in primary sensorimotor cortex when the PD contribution is included in the T1w signal equation. This regional specificity can be interpreted as PD‐related sensitivity to age‐related density reduction of small myelinated fibres in the cortico‐spinal track [Terao et al., 1994]. An alternative explanation accommodating the involvement of sensorimotor rather than other functional areas is based on the idea of specific age‐dependent vulnerability of phylogenetically recent, high‐workload areas related to fine motility of the hands, bipedal locomotion and posture [Ghika, 2008].

Unlike GM volume, there were no differential interactions between cortical thickness and age across synthetic images. This apparent controversy can be explained from both the neurobiological and computational anatomy perspectives. The cortical GM volume variation is linked to differences in thickness and surface area [Storsve et al., 2014]. From a biological point of view, age‐related brain tissue property changes seem to impact mainly surface area rather than cortical thickness [Panizzon et al., 2009]. The relationship between cortical thickness and surface area is very debated: some studies have concluded to the independence [Panizzon et al., 2009] and others to a negative correlation [Winkler et al., 2010] between these two quantities. Regional differences in surface area are driven by cellular events such as synaptogenesis, gliogenesis, intracortical myelination, loss of dendritic size, and complexity [Feldman and Dowd, 1975; Hill et al., 2010]. It has been suggested that intracortical myelination plays a role in the stretching of the cortical surface along the tangential axis [Seldon, 2005]. This stretching is hypothesized to disentangle neighbouring neuronal columns and enable the relevant parts of the cortex to better differentiate afferent signal patterns and increase functional specialization [Hogstrom et al., 2012; Seldon, 2007]. This model provides one possible mechanistic and functional hypothesis explaining the higher GM volume sensitivity towards age‐related changes across synthetic images. Small age‐related PD variations [Callaghan et al., 2014] between gray and white matter, induced by fibers demylination, might then account for the differential age effects detected on GM volumes across synthetic images.

From a methodological point of view, this controversy can be explained by the fact that FreeSurfer uses a fixed model for the intensities of the various tissue classes in T1w scans, whereas SPM estimates the intensity distributions from the images. Accordingly, the FreeSurfer approach can lead to systematic underestimation of the GM tissue class probability in young subjects [Klauschen et al., 2009]. This observation could explain the fact that cortical thickness, estimated by FreeSurfer, is less sensitive in detecting the interaction between age and small contrast variations with respect to GM volume computed by SPM.

### Subcortical Findings

Another important piece of evidence for the profound effect of brain tissue properties on morphological estimates comes from our results in subcortical regions across synthetic image modalities. When including the effects of *R*_2_* in the T1w synthetic images, the GM volume estimates of putamen, globus pallidus and substantia nigra were increased. *R*_2_* is considered a reliable biomarker of iron content which is abundant in the basal ganglia [Aquino et al., 2009; Langkammer et al., 2010]. Similarly to our cortical findings, the modulation of sub‐cortical results by PD appears to be more pronounced in regions where the GM‐WM contrast on the T1w(R1) images is low. In deep brain nuclei this is due the higher iron content that reduces the longitudinal relaxation time [Lorio et al., 2014; Patenaude et al., 2011].

#### Modulation of subcortical findings by age

Our results showing age‐related GM volume decrease in subcortical regions are in agreement with previous studies reporting trends for negative correlation between age and MT‐based GM volume estimates in the dorso‐lateral putamen [Callaghan et al., 2014; Draganski et al., 2011]. Moreover, we show that the inclusion of R2* in the signal equation lowers the negative correlation between GM volume and age. We attribute this effect to the sensitivity of *R*_2_* to iron concentration, which increases with age in the basal ganglia. This contributes to the enhancement of image contrast between gray and white matter, partly counterbalancing the apparent GM decrease appearing in T1w(*R*_1_) images [Hallgren and Sourander, 1958; Stüber et al., 2014; Yao et al., 2009]. We thus demonstrate that increased correlation between GM volume loss and age in subcortical regions is mainly driven by tissue property changes rather than atrophy pattern [Lorio et al., 2014].

### Limitations

We find significant nonlinear age effects on GM volume at the level of the hippocampus and insula, but no significant results on cortical thickness. This might be the result of the narrow separation between the insula or entorhinal cortex and the adjacent putamen or hippocampus, leading to errors in the definition of GM‐WM surface and increasing the variability of thickness estimates [Han et al., 2006]. However the absence of quadratic age effects on cortical thickness is consistent with the literature [Fjell et al., 2009]. Inaccuracies in the reconstruction of the WM or pial surfaces, originating in local misclassification of tissue types, can lead to local erroneous estimations of cortical thickness and depth [Bazin et al., 2014; Lutti et al., 2014]. Since variations in myelination are of the same order along the cortical depth and over the cortical surface, erroneous definition of the WM or pial surfaces can obscure the definition of areal boundaries [Lutti et al., 2014; Waehnert et al., 2016]. Inaccurate surface definition can occur near large pial vessels, or where the gray‐white matter surface closely approaches thin strands of white matter under small gyri, although it should be noted that these comprise only a tiny fraction of temporal cortex.

## CONCLUSION

In this study, we use MRI biomarkers of brain tissue microstructure to investigate the origin of morphological brain changes commonly reported in neuroscience research. We show that myelin, iron and water content yield regionally specific contributions to gray matter volume and cortical thickness estimates obtained from T1‐weighted MRI images. We also demonstrate that associated mechanisms account for a significant fraction of the apparent age‐related gray matter atrophy widely acknowledged in the literature. These results motivate a review of the neurobiological interpretation of previous computational anatomy findings and the use of qMRI biomarkers for the study of the (patho)‐physiological mechanisms in the healthy and diseased brain.

The motivation behind this detailed analysis is to bring to the attention of readers spurious morphological brain changes of microscopic origin that can be detected using T1‐weighted MRI data. We do not recommend systematic use of the approach presented here in future neuroanatomy studies. Rather we highlight the additional value of quantitative MRI for neuroanatomy studies, which provides quantitative and specific biomarkers of tissue microstructure that allow an insight into the biophysical mechanisms underlying brain changes. This motivates the generalization of quantitative MRI over standard anatomical MRI for the study of brain anatomy.

The results presented in this study do not question the validity of morphological measures as markers of brain anatomy. However the choice of MRI data used to extract these measures should be carefully considered. The extraction of morphological measures from qMRI data avoids the limited interpretability of standard anatomical results highlighted here. However, it should be noted that morphological measures obtained from qMRI data remain sensitive to the microstructural property that drive the image contrast. In order to preserve the interpretability of results, we recommend use of a qMRI biomarker specific to the tissue microstructural property of interest. Because of its dominant contribution to the MRI signal, an MRI contrast specific to myelination might be preferred [Helms et al., 2008b, 2009].
